# The effect of suture selection in complex anal fistulas on the success of cutting seton placement and patient comfort

**DOI:** 10.12669/pjms.36.4.1920

**Published:** 2020

**Authors:** Murat Akici, Ogun Ersen

**Affiliations:** 1 Dr. Murat Akici. Department of General Surgery, Afyonkarahisar Health Sciences University, Afyon, Turkey; 2 Dr. Ogun Ersen, Department of General Surgery, Afyonkarahisar Health Sciences University, Afyon, Turkey

**Keywords:** Anal fistula, Seton, Local infection

## Abstract

**Objective:**

The aim of our study was to compare the success rates of suture selection, recovery times and pain associated with local wound infection and seton placement in patients undergoing cutting seton placement for complex anal fistula.

**Methods:**

The study included a total of 90 patients who were admitted with the diagnosis of complex anal fistula between January 2015 and July 2018.

**Results:**

The first session and other revision appointments demonstrated that the number of patients who required fistulotomy was significantly higher in group-1 as the seton failed to complete the transection (p = 0.001). When the patients were asked to rate pain for 3 different conditions according to numeric rating scale (NRS), the patients in group-2 had significantly higher pain in all 3 cases compared to the patients in group-1 (p 0.001). The impact of the suture material on local infection was examined and it was determined that the results of cultures for seton material were significantly more positive in group-1 (p = 0.001).

**Conclusions:**

We conclude that a multi-stage tight seton placement with silk material can lead to satisfactory results by aiming to shorten the cutting time of silk seton.

## INTRODUCTION

Anal fistula is a surgical condition which can be defined as chronic abnormal communication between the epithelialised surface of the anal canal and the perianal skin. Although fistula is detected in 0.71% of patients in the colonoscopy series, the main diagnosis is usually made by anamnesis and physical examination.^1^ Patients present with pain associated with intermittent abscesses or itching and symptoms of anal dermatitis due to discharge.^2^ The disease has an incidence of 1.2-2.8/10.000. The prevalence is 2-fold higher in men than in women.^3^ Although antibiotherapy provides relief in symptoms at the beginning of the disease, the definitive treatment is surgery.^4^ In 1976, Parks et al.^5^ published a groundbreaking classification of anal fistulas with the aim to determine which surgical modality should be preferred. In this classification, the perianal fistulas were divided into four groups such as intersphincteric, transsphincteric, suprasphincteric, and extrasphincteric fistulas based on the distance between the tract and anal sphincter. In addition, there are also studies in the literature which define fistulas as “simple” or “complex” based on the relation between the fistulas and the sphincter. Accordingly, a simple anal fistula includes low transsphincteric and intersphincteric fistulas that cross a maximum of 30% of the external sphincter.^6^ Complex fistulas are defined as suprasphincteric and extra-sphincter fistulas, high trans-sphincter fistulas, horseshoe fistulas and fistulas associated with secondary causes such as radiotherapy, malignancy and inflammatory bowel disease.^7^,^8^ Although various treatment modalities have been defined to preserve the sphincter in complex perianal fistulas, cutting seton is still the most commonly used treatment modality.^9^-^12^ Although this procedure is reliable for anal incontinence, postoperative care and wound healing are laborious and long lasting for patients. Patients usually wait 1 to 2 months for the cutting seton to fall out, which is even prolonged in the event of recurrence. Repetitive operations and defecation problems during the recovery period may lead to social phobia and delay in return to work life. Therefore, a greater number of studies is needed to examine the effect of suture material on outcomes of the surgery in order to carry out excellent seton procedures. Although there are many studies in the literature such as metanalyses comparing different techniques along with the material, retrospective studies or single group studies, there are very few prospective randomized studies including comparisons with the same technique.^13^,^14^ The aim of our study was to compare the success rates of suture selection, recovery times and pain associated with local wound infection and seton placement in patients undergoing cutting seton placement for complex anal fistula.

## METHODS

The study included a total of 90 patients who were admitted to the General Surgery Clinic of Afyon Medical Sciences University with the diagnosis of complex anal fistula between January 2015 and July 2018. An approval (Ref. # 2019/8, dated July 5, 2019) was obtained from the Ethics Committee for the study. Informed consent was obtained from all patients included in the study group. The exclusion criteria included patients undergoing previous surgeries due to anal fistula, patients with abscesses, patients with inflammatory bowel disease and patients with multiple fistulas. The patients were randomized by the surgeon according to the Parks classification using a magnetic resonance imaging method. The patients undergoing 0 silk seton placement were classified as group-1 and the patients undergoing 0 polypropylene suture placement were classified as group-2. The patient demographics, surgery and recurrence data, follow-up time, seton fall-out time and surgical wound infection were recorded in the pre-designed information forms and electronic medical records.

All patients were administered laxative enema to have an empty bowel before surgery. 1g of cefuroxime axetil was applied intravenously for surgical prophylaxis because of the contaminated wound according to ACS-NSQIP surgical wound classification. The patients were operated under general or spinal anesthesia in the lithotomy position. The anus was opened with the help of anoscope and hydrogen peroxide was administered through the external opening. After its exposure, the external opening of the fistula tract was then gently probed till the internal opening, at this time, the length of the fistula tract was measured over the probe wire. The fistula tract up to the sphincter was excised and curated. The suture was then attached to the probe and passed through the sphincter inserting a tight seton using 0 silk suture in group-1 patients and 0 polypropylene suture in group two patients. All surgical procedures were performed by the same surgical team with the same surgical technique. In the postoperative period, patients who had no complications due to surgery or anesthesia were discharged on the following day. They were told to have a sit bath with ethacridine lactate (rivanol sashe) after each defecation and were scheduled for follow-up at 1 month intervals.

At the first follow-up appointment, a 1 cm piece from the end of the seton materials was dissected and sent for anaerobic culture and the e-coli colonies in culture results were compared by the number of colonies per millilitre (colony-forming unit/millilitre (cfu/mL)). Culture results of 100 000 cfu/mL and above were evaluated as positive and 5000 cfu/mL and below as negative.

During the 1-month follow-up period, the absence of reduced discharge or spontaneous seton fall-out were defined as non-healing. Fistulotomy was performed in patients with up to a 1cm of tissue remaining after seton transection, whereas seton revision was performed for tightening of the seton in other patients. In addition, they were advised to apply to the service immediately if the seton fell out. During the 1-month follow-up period, spontaneous seton fall-out or disappeared discharge after fistulotomy were defined as healing. The re-diagnosis of anal fistula in non-symptomatic healed patients was defined as recurrence.

The patients were asked to rate their pain sensations by using the numeric rating scale (NRS). The pain during daily activities, defecation and rest was noted separately. The scale was performed one month after surgery at the first follow-up appointment with the aim to distinguish the pain associated with seton placement from early postoperative pain.

The data were analyzed using the Statistical Package for the Social Sciences 21 (SPSS, Armonk, New York, IL, USA) The variables were presented as mean, minimum-maximum and percentage. A value of p<0.05 was considered statistically significant.

## RESULTS

The study consisted of 90 patients who underwent a tight-cutting seton placement following the diagnosis of complex anal fistula. In Group-1, 11 (24.4%) of the patients were female and 34 (75.6%) were male. The mean age was 45.5 ± 10.2. 37 (82.2%) patients had transsphincteric fistula, 7 (15.5%) had suprasfinkteric fistula and 1 (2.2%) patient had extrasphincteric fistula.

In Group-2, 12 patients were female (26.6%) and 33 (73.4%) were male. The mean age was 46.7 ± 10.9. 38 (84.4%) patients had transsphincteric fistula, 6 (13.3%) patients had suprasfincteric fistula and 1 (2.2%) patient had extrasphincteric fistula. There was no statistically significant difference in the mean age, gender, and distribution of patients according to fistula classification between the groups (p> 0.05). (Table-I)

**Table-I:** Distribution in groups according to the Parks classification.

	Group-1 (n = 45)	Group-2 (n = 45)	p value
Age	45.5 ± 10.2	46.7 ± 10.9	
Gender (M/F)	34/11	33/12	
Transsphincteric fistula	37 (82.2%)	38 (84.4%)	>0.05
Suprasphincteric fistula	7 (15.5%)	6 (13.3%)	
Extrasphincteric fistula	1 (2.2%)	1 (2.2%)	
Duration of operation (min)	22.1 ± 5.2	23.4 ± 4.3	

The duration of operation was determined as 22.1 ± 5.2 minutes in group-1 and 23.4 ± 4.3 minutes in group-2 after the end of anesthesia preparation (p> 0.05). (Table-I)

The length of hospital stay was 1 day in 83 patients (92.2%). Of 7 patients with a hospital stay longer than one day, four patients were followed up for pain palliation and 3 patients were followed up for additional diseases (COPD, congestive heart disease, asthma) resulting in a prolonged hospital stay.

The comparison of the groups according to healing and non-healing status revealed that the number of healed patients with spontaneous seton fall-out in a single seton session were similar in both groups (p> 0.05). In addition, the first session and other revision appointments demonstrated that the number of patients who required fistulotomy was significantly higher in group-1 as the seton failed to complete the transection (p = 0.001). The number of operations required for healing was also significantly higher in group-1 patients using silk seton than group-2 patients using polypropylene seton (p = 0.001). The mean duration of seton placement showed no significant difference between the patients (p> 0.05). There was no significant difference in the number of recurrence (p> 0.05). The mean follow-up time was 24.2 months. (Table-II)

**Table-II:** Surgery and recurrence data.

	Group-1	Group-2
Healing in one session (n)	20 (44.4%)	23 (51.1%)
Number of patients requiring fistulotomy (n)	23 (51.1%)	12 (26.6%)
Number of operations required for healing (n)	2.6	1.9
Mean duration (days) of seton placement in each session	48.2 ± 5.1	45.3 ± 4.6
Number of recurrent patients (n)	6 (13%)	4 (8.8%)
Mean follow-up time (months)	24.6	

When the patients were asked to rate pain for 3 different conditions according to NRS, the patients in group-2 had significantly higher pain in all 3 cases compared to the patients in group-1 (p 0.001). (Table-III)

**Table-III:** NRS scores by groups.

	Group-1	Group-2	p value
Daily Activity	3.4	6.9	0.001
Defecation	4.5	6.2	
Resting	2.7	4.3	

The impact of the suture material on local infection was examined and it was determined that the results of cultures for seton material were significantly more positive in group-1 patients than in group-2 patients (p = 0.001). The number of negative cultures was significantly higher in group-2 patients (p = 0.001). In group-1, 12 (26.6%) patients had local wound infection accompanied by hyperemia and purulent discharge at the wound site, whereas this number was 10 (22.2%) in group-2. However, there was no statistically significant difference (p> 0.05). (Table-IV)

**Table-IV:** Culture results and wound infection rates by groups.

	Group-1	Group-2	p value
>100,000 cfu/mL e-coli colonies	21 (46.6%)	16 (35.5%)	0.001
<5000 cfu/mL e-coli colonies	5 (11.1%)	12 (26.6%)	0.001
Local wound infection	12 (26.6%)	10 (22.2)	>0.05

## DISCUSSION

Setons are mostly used either as a loose seton or as a tighter cutting seton. Loose seton placement is usually performed for drainage in order to prevent the obstruction of the fistula tract due to chronic inflammatory diseases in the anal region and inflammatory bowel diseases. The perianal fistulas caused by other reasons constitute the majority of patients and they are treated with cutting seton. The correct selection of seton materials is important to ensure the quality of life of patients at a sustainable level and provide healing with a minimum number of operations possible. Silk and nylon sutures, metal wire, elastic band, penrose drain, pieces of surgical gloves, plastic clamps are among the seton materials reported to be used in the literature, but the majority prefer surgical sutures.^15^ In 1976, Parks and Stitz reported their results on the use of nylon surgical sutures for seton placement. Then, in 2002, a case series of 47 patients undergoing polypropylene PP seton placement reported that the mean seton duration was 9 weeks with a 2% of recurrence rate.^6^,^16^ Another study of 30 patients reported that 80% of the patients who underwent cutting seton placement were healed in 5-10 weeks.^17^ In 2002, a study conducted by Durgun et al. reported no recurrence in 10 patients and the mean waiting time was 40 days.^18^ The waiting period was reported as 14 months in a retrospective series of 24 patients undergoing tight seton placement.^19^ The mean wound healing time was reported as 8 weeks in 68 patients who underwent silk cutting seton placement.^20^ 75% of the patients stated that wound healing was completed in 8 weeks, but the seton fall-out time was not presented as in any of the other studies.^21^ The reason for long duration in this study was attributed to the fact that 25% of the study population consisted of patients with Crohn’s disease. Subhas et al. carried out a meta-analysis compiling the literature results which compared the outcomes related to each material, however, no definite conclusion was reached because of the differences in the surgical techniques.^21^

Although there was no significant difference in the outcomes, it was determined that the number of patients healed in a single session was similar in both groups using silk and PP sutures, however, the number of patients requiring multiple sessions was higher in the silk suture group. In addition, it was observed that the requirement for fistulotomy was quite high in most patients undergoing silk seton placement as it failed to transect after a certain level. It was also demonstrated that the pain and discomfort were significantly higher in group-2 patients than in group-1 even though the PP material provided a better cut. The results revealed that PP seton caused a significantly higher amount of pain compared to silk seton especially during daily activities, which was persistent even during defecation and rest.

Banche et al.^22^ examined the effect of various suture materials on bacterial adhesion in the mouth. In the study, they evaluated 5 groups of suture materials such as silk, Supramid, Synthofil, Ethibond Excel, Ti-cron Monocryl inserted in 60 patients undergoing dentoalveolar surgery. Most bacterial adhesion was observed in silk. In conclusion, it was reported that the suture materials inserted in the oral cavity should be taken out as soon as possible.

In the literature, there are no studies showing the effect of suture materials on bacterial adhesion in the perianal region. Consistent with the study of Banche et al., our study revealed significantly higher e-coli colonization in the silk seton group. We think that it is because the polypropylene and irregular surface of the silk yarn facilitates bacterial colonization. The wound site was similar in both groups unlike the culture results with regard to the local wound infection and discharge in patients undergoing silk seton and PP seton placement. We see that silk seton is predisposed to bacterial colonization but it is not reflected in the clinical outcomes.

### Limitations of the study

The limitations of our study are that the sample size is not large enough and the groups are not randomized. In addition, evaluation of only e-coli colonization in culture results shows fecal contamination adequately, but for optimal results, it is necessary to conduct studies considering other pathogens.

## CONCLUSION

It has been concluded that patients using silk sutures undergo a greater number of surgeries averagely, which results in a longer duration of seton placement. However, it has been observed that the level of comfort is much higher in the silk group than in the PP group. There was no difference in wound infection and recurrence between the two groups. In the light of these data, it may be appropriate to say that patients undergoing silk seton placement may need longer and repetitive procedures, whereas patients undergoing PP suture placement may experience persistent pain and discomfort in the following postoperative days. We think that a multi-stage tight seton placement with silk material can lead to satisfactory results by aiming to shorten the cutting time of silk seton.

### Authors’ Contribution

**MA:** Conceived, designed, did statistical analysis & editing of manuscript.

**MA, OE:** Did data collection and manuscript writing.

**MA, OE:** Did review and final approval of manuscript.

**MA:** Is responsible for the accuracy and integrity of the work.

## References

[1] Ozsoy M, Celep B, Ersen O, Ozkececi T, Bal A, Yilmaz S (2014). Our results of lower gastrointestinal endoscopy:evaluation of 700 patients. Turk J Surg (Ulusal Cerrahi Dergisi).

[2] Tozer P, Sala S, Cianci V, Kalmar K, Atkin GK, Rahbour G (2013). Fistulotomy in the tertiary setting can achieve high rates of fistula cure with an acceptable risk of deterioration in continence. J Gastrointest Surg.

[3] Zanotti C, Martinez-Puente C, Pascual I, Pascual M, Herreros D, Garcia-Olmo D (2007). An assessment of the incidence of fistula-in-ano in four countries of the European Union. Int J Colorectal Dis.

[4] Ersen O, Akici M, Çelik G (2019). Is fistulotomy adequate in the treatment of simple perianal fistula?. Med J SDU.

[5] Parks AG, Gordon PH, Hardcastle JD (1976). A classification of fistula-in-ano. Br J Surg.

[6] Parks AG, Stitz RW (1976). The treatment of high fistulain-ano. Dis Colon Rectum.

[7] Galis-Rozen E, Tulchinsky H, Rosen A, Eldar S, Rabau M, Stepanski A (2010). Long?term outcome of loose seton for complex anal fistula:a two?centre study of patients with and without Crohn's disease. Colorectal Dis.

[8] Sangwan YP, Rosen L, Riether RD, Stasik JJ, Sheets JA, Khubchandani IT (1994). Is simple fistula-in-ano simple?. Dis Colon Rectum.

[9] Demirel AH (2016). The cutting seton technique with the tractus medialisation in the long tractus anal fistula (New technical description). J Surg Arts.

[10] Ortiz H, Marzo J (2000). Endorectal flap advancement repair and fistulectomy for high transsphincteric and suprasphincteric fistulas. Br J Surg.

[11] Bleier JI, Moloo H, Goldberg SM (2010). Ligation of the intersphincteric fistula tract:an effective new technique for complex fistulas. Dis Colon Rectum.

[12] Leventoglu S, Mentes BB (2007). Anal Fistula Plug for Treatment of Complex Anorectal Fistula. Kolon Rektum Hast Derg.

[13] Amato A, Bottini C, De Nardi P, Giamundo P, Lauretta A, Luc AR (2020). Evaluation and management of perianal abscess and anal fistula:SICCR position statement. Tech Coloproctol.

[14] Göttgens KWA, Smeets RR, Stassen LPS, Beets G, Breukink SO (2015). Systematic review and meta-analysis of surgical interventions for high cryptoglandular perianal fistula. Int J Colorectal Dis.

[15] Williams JG, MacLeod CA, Rothenberger DA, Goldberg SM (1991). Seton treatment of high anal fistulae. Br J Surg.

[16] Theerapol A, So BY, Ngoi SS (2002). Routine use of setons for the treatment of anal fistulae. Singapore Med J.

[17] El Mohamady MS (2018). Cutting seton in management of complex perianal fistula–is it a safe procedure?. Menoufia Med J.

[18] Durgun V, Perek A, Kapan M, Kapan S, Perek S (2002). Partial fistulotomy and modified cutting seton procedure in the treatment of high extrasphincteric perianal fistulae. Dig Surg.

[19] Subhas G (2011). Non-cutting setons for progressive migration of complex fistula tracts:a new spin on an old technique. J Colorectal Dis.

[20] Al-Marzooq TJ, Hassan QA, Alnaser MK, Al-Edani MS (2017). Treatment of Fistula-In-Ano with Tight (Cutting) Seton:Analysis of Outcomes and Efficacy Assessment. J Clin Diag Res.

[21] Subhas G, Bhullar JS, Al-Omari A, Unawane A, Mittal VK, Pearlman R (2012). Setons in the treatment of anal fistula:review of variations in materials and techniques. Digest Surg.

[22] Banche G, Roana J, Mandras N, Amasio M, Gallesio C, Allizond V (2007). Microbial adherence on various intraoral suture materials in patients undergoing dental surgery. J Oral Maxillofac Surg.

